# 2373: Effects of cortical stimulation of the noninfarcted Versus peri-infarcted motor cortex

**DOI:** 10.1017/cts.2017.45

**Published:** 2018-05-10

**Authors:** Serena-Kaye Kinley-Cooper, DeAnna Adkins

## Abstract

OBJECTIVES/SPECIFIC AIMS: The objectives of this study are to determine whether high-frequency ipsi-lesion or low-frequency contra-lesion ECS improves forelimb function following experimental stroke in aged animals with focal and large strokes. We also want to investigate whether ECS-induced improvements in motor function are related to an enhancement of neural structural plasticity (dendrites and synapses) and changes in growth promoting (BDNF) and growth inhibiting (NOGO-A) expression in the infarcted motor cortex in young and aged animals. METHODS/STUDY POPULATION: We will investigate whether excitatory ECS of the infarcted cortex or inhibition of the noninfarcted cortex combined with daily impaired-forelimb rehabilitative training (RT) results in greater motor functional recovery compared to RT alone. Immunohistochemical (IHC) analyses and unbiased stereological techniques will be performed to investigate changes in proteins associated with dendritic restructuring (MAP2), synaptic plasticity (PSD95 and synaptophysin), and alteration in the expression of BDNF and NOGO-A. RESULTS/ANTICIPATED RESULTS: We expect that inhibitory ECS of the noninfarcted motor cortex will improve behavioral outcomes in moderate to severe stroke animals compared with excitatory ECS or no stimulation (RT alone) animals. We predict that the ECS condition that improves motor performance most significantly compared with RT alone will have a corresponding greater increase in remaining ipsi-infarct motor cortical dendritic and synaptic plasticity (demonstrated by a greater density of MAP2, synaptophysin, and PSD-95 immunoreactivity), and greater expression of BDNF. It is unknown, but also expected that better behavioral recovery will coincide with a greater reduction in NOGO-A in the injured motor cortex. DISCUSSION/SIGNIFICANCE OF IMPACT: These studies will aid in creating a model that will allow for a better understanding of the relationship between brain stimulation, severity of injury and, in future studies, aging. These studies will also help clarify previous conflicting brain stimulation results.

